# Prevalence of *Schistosoma mansoni* Infection and Associated Risk Factors among School Children in Jiga Town, Northwest-Ethiopia: A Cross-Sectional Study

**DOI:** 10.1155/2020/6903912

**Published:** 2020-06-26

**Authors:** Ketemaw Wubet, Destaw Damtie

**Affiliations:** ^1^Finote Selam Teachers College, Finote Selam, Ethiopia; ^2^Department of Biology, College of Sciences, Bahir Dar University, Bahir Dar, Ethiopia

## Abstract

**Background:**

Schistosomiasis, one of the 20 Neglected Tropical Diseases, is a human disease which is caused by infection with *Schistosoma* spp. In Ethiopia, the intestinal form of schistosomiasis (caused by *Schistosoma mansoni*) is widely distributed. This study is aimed at assessing the prevalence of *S. mansoni* and associated risk factors among schoolchildren in Jiga town and its surrounding rural “Kebeles”, Northwest Ethiopia (Kebele = the smallest administrative unit in Ethiopia).

**Methods:**

A cross-sectional study was carried out among children from three schools in Jiga town and its surrounding rural “Kebeles”. The schools were selected mainly according to their proximity to water bodies that were likely to have been used by the children. A total of 362 randomly selected children were screened for *S. mansoni* infection. Samples of stool were collected from each pupil and examined by the concentration and microscopic techniques. Semistructured interview questionnaires were administered to the study subjects to identify the possible risk factors of infection with *S. mansoni*. Data analysis was performed using SPSS version 20 software.

**Results:**

The overall prevalence of *S. mansoni* was 15.2%. A higher proportion of *S. mansoni* infection (30.3%) was observed at Chifarit primary school children followed by Tikur Wuha primary school children (17.3%) and Jiga 01 primary school children (10.2%). The habit of contact with river water (AOR, 4.86; 95% CI, 0.991-23.8; *P* = 0.051) and the irregular wearing of shoes (AOR, 6.06; 95% CI, 2.61-14.13; *P* = 0.001) were risk factors for *S. mansoni* infection.

**Conclusion:**

*S. mansoni* infection remains a public health problem in the study area. Snail control, mass deworming, and health promotion are recommended.

## 1. Background

Schistosomiasis, one of the 20 Neglected Tropical Diseases (NTDs) (WHO) [1], is a human disease which is caused by infection with Schistosoma spp. Schistosomes use mammalian definitive hosts and freshwater snail intermediate hosts [2]. Schistosomiasis has been reported from 78 countries. In 2017, over 220.8 million people required preventive treatment 90% of whom were from Africa [3]. About 13.6% of the risk group lives in irrigation schemes. The majority of *S. mansoni* infections occur in Sub-Saharan Africa [4]. In Ethiopia, S. *mansoni* is widely distributed [5]. About 5.01 million people are thought to suffer from schistosomiasis and 37.5 million are at risk of getting the infection [6].

Globally, schistosomiasis results in 3.3 million disability-adjusted life years (DALYs). Most infected people live in poor communities without access to safe drinking water and adequate sanitation. An estimated 90% of infected individuals live in Sub-Saharan Africa, where up to 20 million people also suffer severe chronic health consequences of the disease [7]. In children, schistosomiasis can cause anemia, stunting, and a reduced ability to learn, although the effects are usually reversible with treatment [3]. Elimination of the disease is possible by mass drug administration with 40 mg/kg of praziquantel and integrated control targeting the life cycle [8].

Human infection happens due to exposure to cercariae containing water [9, 10]. Transmission of Schistosomes relies on contamination of water by excreta, intermediate hosts (snails), and skin exposure to contaminated water [11].

Social, cultural, behavioral, and economic factors interact with local environmental and ecological factors to produce extraordinary variation in the epidemiology of schistosomiasis concerning the prevalence and intensity of infection [2]. Human behaviors such as swimming habits, improper waste disposal, use of river water for different purposes, and wide distribution of intermediate hosts are the major contributing factors for the increased prevalence and wide distribution of schistosomiasis [10].

According to Nute et al. [12], the overall prevalence of *S. mansoni* in the Amhara Region was 6.9%. Schistosomiasis prevalence in the study area in 1986 was 37.7% [13]. The presence of Tikur Wuha irrigation dam, irrigation canals to Chifarit and Tikur Wuha “Kebeles”, and wetlands in these “Kebeles” are fertile grounds for *S. mansoni* infection. However, recent studies have not been conducted in the area. Therefore, this study was aimed at investigating the prevalence and associated risk factors of schistosome infection among school children from three primary schools in Jiga town and its surrounding rural “Kebeles”, Ethiopia.

## 2. Methods

### 2.1. Study Design and Period

A cross-sectional study was conducted at the Jiga Health Center from November 2018 to March 2019, among students from three schools (Tikur Wuha, Chifarit, and Jiga 01 primary Schools).

### 2.2. Study Area

The study was carried out among school children in Jiga town and the surrounding rural “Kebeles”, Amhara Region, 372 km away from Addis Ababa. Jiga town is characterized by; 1850 m altitude, 1000 mm mean annual rainfall, and 18-28°C temperature range. Three rivers, Tikur Wuha, Giligle Tikur Wuha, and Laza, flow through the study area. Tikur Wuha River is used for bathing, cloth washing, irrigation, recreation, source of tap water, and other domestic activities. According to the Central Statistical Agency (CSA) [14], the town has a total population of 4612 (2217 males and 2395 females). Social service institutions in the town are five private clinics, one major health center, and six schools. A total of 4383 (2160 male, 2223 female) students were enrolled in the three schools in the 2018/2019 academic year.

### 2.3. Study Population

The study population was students of Tikur Wuha, Chifarit, and Jiga 01 primary Schools who were enrolled in the study period.

### 2.4. Sampling and Sample Size Determination

The sample size was determined using the following formula [15]:

(1) $$\begin{array}{c} n=\frac{\text{z}2\;p\;\left(1-P\right)/e2}{1+\left(z2\;p\;\left(1-p\right)/e2N\right)} \end{array}$$

where *n* = sample size, *N* = population size (4383), *z* = *z*-score (1.96), *e* = margin of error (0.05), and *p* = population proportion (37.7%) [13]. The computed sample size was 334. Considering some nonresponse rate, 10% of the samples (33) were added resulting in a total sample size of 367. Furthermore, an online sample size calculator was employed, and the result was the same [16]. The three schools were selected based on their proximity to a river or bodies of water where snails, suspected to be the vector of schistosomiasis in the area, are present and where children are often seen swimming. Students from these schools were expected as they are more vulnerable to schistosome infection than other schools in the town. Students were selected by stratified random sampling techniques using the school of students as a sampling frame. The number of sample students from each stratum (school) was determined using a proportion allocation formula *n* = *p*^∗^*N*, where, *p* = proportion, *N* = total sample size. Based on this formula the calculated number of sample students in each school was as follows; Chifarit = (402/4383)367 = 34, Tikur Wuha = (1960/4383)367 = 164, Jiga 01 = (2021/4383)367 = 169. The name of all students from grade 1-8 in each school was listed down and arranged alphabetically in separate excel sheets. Then, participant students were selected randomly from each school.

### 2.5. Sociodemographic Characteristics and Associated Risk Factors

Sociodemographic, behavioral, and risk factor data were collected using a structured interview of the questionnaire. The questionnaire was prepared in English and translated to the Amharic language for simplicity and translated back to English to check the consistency.

### 2.6. Sample Collection and Processing

About 2 g of fresh stool specimen was taken from each participant using dry, clean, and prelabeled containers. Stool samples were immediately processed and analyzed. *S. mansoni* eggs were obtained and identified using the concentration and microscopy methods. The community category of schistosome prevalence was classified as low (<10%), medium (10-50%), and high (>50%) (WHO) [17].

### 2.7. Data Management and Statistical Analysis

The data collected were encoded and analyzed using SPSS version 20. Descriptive statistics and logistic regression were used to determine the prevalence of *S. mansoni* infection and its association with risk factors. Bivariate and multivariate logistic regression analysis was used to estimate Crude OR and adjusted OR, respectively, at 95% Confidence Interval (CI). The analysis was made based on age and grade level because of maturity and understanding differences across grade levels and age groups.

### 2.8. Laboratory Procedure

Both the direct wet mount microscopy and formol-ether concentration (FEC) methods were performed in the Jiga health center laboratory. The test procedures were carried out following standard protocols reported by World Health Organization (WHO) [18].

## 3. Results

### 3.1. Sociodemographic Characteristics

A total of 362 children participated in this study (219 males, 143 females). The mean age of the participants was 12.5 years. The majority (52.8%) lay in the grade levels of 5–8. The residence of study participants showed that 61.9% of them were urban dwellers. The parents of the majority of the study participants were farmers; 168 (46.4%) and 118 (59.0%) of the children had poor knowledge about the correct modes of transmission of schistosomiasis (Table 1).

### 3.2. Behavioral and Environmental Factors for *S. mansoni*Infection

Respondents who used to wear shoes usually, regularly, and sometimes were; 43.1%, 33.4%, and 23.5%, respectively. The respective proportions of toilet users and nonusers were 55% and 45%. Most of the respondents (58.3%) reported swimming in rivers and 98.1% of them had contact with river waters. A significant proportion of the participants (34.8%) were using river/well waters for drinking (Table 2).

### 3.3. Prevalence of *S. mansoni* Infection by Sociodemographic, Environmental, and Behavioral Factors

The overall prevalence of *S. mansoni* infection was 55 (15.2%). Based on the bivariable logistic regression, the age groups 7 to 10 (COR, 3.83; 95% CI, 1.554-9.466; *P* = 0.004) and 11 to 14 (COR, 3.465; CI, 1.449-8.284; *P* = 0.005), grade levels 1 to 4 (COR, 2.200; 95% CI, 1.215, 3.984; *P* = 0.009), and the school (Chifarit) (COR, 3.836; CI, 1.556-9.398, *P* = 0.003) were risk factors for *S. mansoni* infection (Table 3). However, in the multivariable logistic regression, the habit of contact with river water (AOR, 4.86; 95% CI, 0.991-23.8; *P* = 0.051) and the irregular wearing of shoes (AOR, 6.06; 95% CI, 2.61-14.13; *P* = 0.001) were risk factors for *S. mansoni* infection (Table 3).

## 4. Discussion

The prevalence of *S. mansoni* infection (15.2%) was medium [17]. This agreed with the reports from Tanzanian (15.1%) and Nigerian (17.8%) [19] and reports in Ethiopia; 20.6% [10], 15-20% [20], and 18.9% [21]. However, it was lower than a report from the same area (37.7%) [13]. This difference might be due to snail control, chemotherapy, health education, and environmental management and modification [22]. It too may be a result of the country's National Neglected Tropical Disease (NTD) Master Plan which was launched in June 2013. As of September 2015, the Federal Ministry of Health has managed to mobilize support to implement mass drug administration in 100% of the endemic districts for schistosomiasis [23].

Similarly, the prevalence of *S. mansoni* infection in the present study was lower than the findings in Tanzania (63.5%) [19], in Egypt (60%) [24], in Zimbabwe (50%), and other findings in Ethiopia such as 37.5% [2] and 24% [25]. Schistosome prevalence in the present study was also much lower than 89.9% [26], 82.8% [27], 40.1% [12], and 24.9% [28]. These variations may be due to variations in diagnosis methods, water velocity, lifestyles [26], local endemicity of the parasite [26], and temperature [28].

On the contrary, the schistosome prevalence of the present study (15.2%) was higher than the prevalence in Egypt (6%) [24]; the regional (36.4%) and zonal (0.5 to 40.1%) prevalences [12], and among Kindergarten Children in Mekelle Town (0.25%) [29]. This variation might be due to variations in sociodemographic factors, immune status of participants, distance from the water source and frequency of contact with infected water, ecological distribution of intermediate host (snail), local endemicity of the parasite, sample size, and awareness regarding the transmission and prevention of *S. mansoni* infection [2].

The age groups 7 to 10 years were more infected with *S. mansoni* than the 11 to 14 and ≥15-year-olds. This finding was in agreement with the findings of [4, 21, 27]. The reason why children aged from 7 to 10 years were more likely to have *S. mansoni* infection might be because children of these age groups are naturally active and have a strong affinity for water-related activities and the responsibility to look after cattle in wetland areas. On the other hand, the relatively low prevalence of *S. mansoni* infection among school children in the age group between 11 to 14 and ≥15 might be due to praziquantel drug mass deworming activities for school children [30]. However, the age-specific result of the present study was in contrast to the findings reported by researchers [20, 31].

The other significant factor associated with *S. mansoni* infection in this study was the grade level of the study participants. Students with grade levels of 1 to 4 were more likely to be exposed to *S. mansoni* infection than grade levels 5 to 8. The possible reason may be a lower level of awareness about personal hygiene and the mode of *S. mansoni* transmission among children whose grade levels were 1 to 4. This finding was in agreement with the findings of [4, 10]. But it disagreed with the findings of [20, 26].

In the school-specific prevalence of *S. mansoni* infection, the highest prevalence was observed in Chifarit primary school (30.3%), followed by Tikur Wuha (17.1%), Jiga 01 primary school (10.6%). This variation could be due to continuous water contact habits and barefoot irrigation activities. All participants in Chifarit School came from farmer families. In their village and near to their school, there is irrigation canal water. They used this water for different purposes like cloth and body washing, cleaning utensils, to grow vegetables, and to clean their classrooms. They cross the water when they come to school and go to their homes. There is also a wetland near their school and villages.

The prevalence of *S. mansoni* infection among female and male participants was 15.4% and 15.1%, respectively, but no statistical difference was observed. This agrees with the findings of [4, 26] but disagrees with the findings of [2, 10, 21].

In this study, students who used to wash their clothes in a river or irrigation canal water and had a habit of taking bath in a river were more likely to acquire *S. mansoni* infection and the difference was statistically significant (*P* = 0.008). This is supported by the study conducted by [10, 21, 26, 27]. Swimming, washing clothes in rivers, and bathing in river waters were reported to be significantly associated with a high risk of *S. mansoni* infection [21]. Likewise, the findings in the present study showed that students who had the habit of swimming in rivers and irrigation canals were more likely to acquire *S. mansoni* infection and the difference was statistically significant (*P* = 0.005). This coincides with reports by [4, 21, 26, 27, 31].

In the present study, there was variation in *S. mansoni* infection related to the habit of shoes wearing among the participant school children. Participants who wore shoes sometimes were 5.34 times more likely for *S. mansoni* infection than participants who wore shoes regularly. This variation was statistically significant (*P* = 0.001) and agrees with research findings reported from School Children in Sanja Town [26] and School Children in Mana District [31]. Besides, participants from Chifarit primary school wore shoes sometimes since there is a wetland in their village. Especially, during the summer season, the wetland was not suitable to wear shoes. This could increase the probability of school children to be infected with *S. mansoni*. Similarly, the habit of shoes wearing among school children was one of the most risk factors for the prevalence of *S. mansoni* in the study area.

## 5. Conclusion

The overall prevalence of *S. mansoni* infection among students from the three schools was medium (15.2%). The highest prevalence was registered among Chifarit primary school students (30.3%) followed by Tikur Wuha primary school (17.1%) and Jiga 01 primary school (10.6%) in descending order. Grades 1-4, ages 7 to10 and 11 to 14, Chifarit primary school, contact with river water, and the irregular wearing of shoes were risk factors for *S. mansoni* infection.

### 5.1. Limitation of the Study

Not using Kato-Katz slides as a golden standard may underestimate the results.

## Figures and Tables

**Table 1:** Sociodemographic distribution among school children in Jiga town and its surrounding rural “Kebeles”, Amhara Region, Ethiopia, March 2019 (*n* = 362).

Sociodemographic characteristics	Frequency	Percent
Sex		
Male	219	60.5
Female	143	39.5
School name		
Chifarit	33	9.1
Tikur Wuha	162	44.8
Jiga 01	167	46.1
Grade level		
1–4	171	47.2
5-8	191	52.8
Age in year		
7–10	104	28.7
10-14	145	40.1
≥15	113	31.2
Residence		
Jiga town	224	61.9
Rural villages	138	38.1
Parents occupation		
Farmers	168	46.4
Merchants	130	35.9
Gov. employee	64	17.7
Knowledge about schistosomiasis		
Yes	86	23.8
No	276	76.2

**Table 2:** Study participants' responses on associated risk factors among school children in Jiga town and its surrounding rural “Kebeles”, March 2019, (*n* = 362).

Associated risk factors	Frequency	Percent
Shoes wearing habit		
Sometimes	85	23.5
Usually	156	43.1
Regularly	121	33.4
Use toilet		
Yes	199	55
No	163	45
Swimming in river water		
Yes	211	58.3
No	151	41.7
Contact with river water		
Yes	355	98.1
No	7	1.9
Drinking water source		
Tap	223	61.6
River/well	139	34.8

**Table 3:** Prevalence and odds ratios (OR) of *S. mansoni* infection by sociodemographic, environmental, and behavioral actors of school children in Jiga town and its surrounding rural “Kebeles” (2019).

Determinant factor	*S. mansoni*		*P* value	COR (95% CI)	*P* value	AOR (95%)
	Positive	Negative				
Age of participants						
7-10	21 (20.2)^@^	83 (79.8)	0.004^∗^	3.831 (1.554-9.446)	0.205	2.389 (0.621,9.19)
11-14	27 (18.6)	118 (81.4)	0.005^∗^	3.465 (1.449, 8.284)	0.018	3.04 (1.12, 8.21)
≥15	7 (6.2)	106 (93.8)		1		1
Grade level						
1-4	35 (20.5)	136 (79.5)	0.009^∗^	2.200 (1.215, 3.984)	0.371	1.849 (0.482, 7.10)
5-8	20 (10.5)	171 (89.5)		1		1
School of participants						
Chifarit	10 (30.3)	23 (69.7)	0.003^∗^	3.836 (1.556, 9.398)	0.646	1.491 (0.272, 8.18)
T/Wuha	28 (17.3)	134 (82.7)	0.063	1.844 (0.966, 3.518)	0.513	0.629 (0.150, 2.50)
Jiga 01	17 (10.2)	150 (89.8)		1		1
Knowledge about schistosomiasis						
Yes	8 (9.3)	78 (90.7)	0.079	1	0.613	1
No	47 (17)	229 (83)		2.036 (0.922, 4.495)		0.621 (0.149,2.43)
Sex of participants						
Male	33 (15.1)	186 (84.9)	0.935	1.025 (0.570, 1.840)		
Female	22 (15.4)	121 (84.6)		1		
Residence						
Jiga town	21 (15.1)	118 (84.9)		1		
Rural villages	34 (15.2)	189 (84.8)	0.971	0.989 (0.548, 1.786)		
Parents occupation						
Farmer	32 (19.1)	136 (80.9)	0.157	1.882 (0.785, 4.516)		
Merchants	16 (12.2)	115 (87.8)	0.824	1.113 (0.433, 2.860)		
Gov't employee	7 (11.1)	56 (88.6)		1		
River water contact						
No	2 (28.6)^@^	5 (71.4)		1		1
Yes	53 (14.9)	302 (85.1)	0.008^∗^	7.948 (1.728, 36.559)	0.051	4.86 (0.991, 23.8)
Shoes wearing habit						
Sometimes	21 (24.7)	64 (75.3)	<0.001^∗^	5.344 (2.154, 13.256)	0.001^∗∗^	6.06 (2.6, 14.13)
Usually	27 (17.3)	129 (82.7)	0.006^∗^	3.409 (1.403, 8.125)		
Regularly	7 (5.8)	114 (94.2)		1		1
Proper latrine usage						
No	27 (16.6)	136 (83.4)	0.511	1.121 (0.683, 2.154)		
Yes	28 (14.1)	171 (85.9)		1		
Swimming in river water						
No	13 (8.7)	136 (91.3)		1		1
Yes	42 (19.7)	171 (80.3)	0.005	0.389 (0.201, 0.754)	0.997	
Crossing a river with barefoot						
No	21 (14.4)	125 (85.6)		1		
Yes	34 (15.7)	182 (84.3)	0.724	0.899 (0.499, 1.622)		
Drinking water source						
Tap	27 (12.1)	196 (87.9)		1		1
River/well	28 (20.1)	111 (79.9)	0.040^∗^	1.831 (1.028, 3.263)	0.998	

Note: ^@^No (%), ^∗^ statistically significant at *P* < 0.

## Data Availability

All datasets generated and analyzed during the study are presented in the paper.

## Abbreviations

AOR: Adjusted odds ratio
BDU: Bahir Dar University
CDC: Center for Disease Control
CI: Confidence interval
COR: Crude odds ratio
CSA: Central Statistics Agency of Ethiopia
NTDs: Neglected tropical diseases
Spp: Species
SPSS: Statistical package for social science
WHO: World Health Organization.
